# Determinants of unsuccessful tuberculosis treatment outcome in Northern Red Sea region, Eritrea

**DOI:** 10.1371/journal.pone.0273069

**Published:** 2022-08-15

**Authors:** Zenawi Zeramariam Araia, Fitsum Kibreab, Abiel Abraham Kibrom, Amanuel Hadgu Mebrahtu, Michael Goitom Girmatsion, Yonatan Woldu Teklehiwet, Araia Berhane Mesfin

**Affiliations:** 1 National TB and Leprosy Control Program, Ministry of Health, Asmara, Eritrea; 2 Human Resource Development, Planning and Policy, Ministry of Health, Asmara, Eritrea; 3 TB and Leprosy Control Program, Northern Red Sea Region, Massawa, Eritrea; 4 Afabet Hospital, Afabet, Eritrea; 5 Nakfa Hospital, Nakfa, Eritrea; 6 Communicable Diseases Control, Ministry of Health, Asmara, Eritrea; Fundació Institut d’Investigació en Ciències de la Salut Germans Trias i Pujol, Universitat Autònoma de Barcelona, SPAIN

## Abstract

**Background:**

Eritrea has achieved the global target (90%) for tuberculosis (TB) treatment success rate. Though, events of unsuccessful TB treatment outcomes (death, treatment failure, lost to follow up and not evaluated) could lead to further TB transmission and the development of resistant strains. Hence, factors related to these events should be explored and addressed. This study aims to fill the gap in evidence by identifying the determinants of unsuccessful TB treatment outcomes in Eritrea’s Northern Red Sea region.

**Methods:**

A retrospective cohort study was conducted in Eritrea’s Northern Red Sea region. Data collected using a data extraction tool was analyzed using Stata version 13. Frequencies, proportions, median and standard deviations were used to describe the data. Furthermore, univariable and multivariable logistic regression analysis were performed to determine the risk factors for unsuccessful TB treatment outcomes. Crude odds ratio (COR) and adjusted odds ratio (AOR) with their 95% confidence interval (CI) presented and p-value < 0.05 was considered statistically significant.

**Results:**

Among 1227 TB patients included in this study, 9.6% had unsuccessful TB treatment outcomes. In multivariable logistic regression analysis, TB cases 55–64 years old (AOR: 2.75[CI: 1.21–6.32], p = 0.016) and those ≥ 65 years old (AOR: 4.02[CI: 1.72–9.45], p = 0.001) had 2.7 and 4 times higher likelihood of unsuccessful TB treatment outcome respectively. In addition, HIV positive TB patients (AOR: 5.13[CI: 1.87–14.06], p = 0.002) were 5 times more likely to have unsuccessful TB treatment outcome. TB treatment in Ghindae Regional Referral Hospital (AOR: 5.01[2.61–9.61], p < 0.001), Massawa Hospital (AOR: 4.35[2.28–8.30], p< 0.001) and Nakfa Hospital (AOR: 2.53[1.15–5.53], p = 0.021) was associated with 5, 4 and 2.5 higher odds of unsuccessful TB treatment outcome respectively.

**Conclusion:**

In this setting, old age, HIV co-infection and health facility were the independent predictors of unsuccessful TB treatment outcome.

## Introduction

Tuberculosis (TB) remains a major global health problem, causing ill health for nearly 10 million people yearly and is one of the top ten causes of death worldwide. For the past five years, it has been the primary cause of mortality from a single infectious agent, standing above Human Immune deficiency Virus/Acquired Immune Deficiency Syndrome (HIV/AIDS) [1].

Early diagnosis of TB accompanied by appropriate treatment prevents death, reduces transmission in the population and contributes to controlling and eliminating TB [1, 2]. Globally, between 2000 and 2019 alone, TB treatment has saved the lives of 63 million TB patients [1]. Hence, monitoring and evaluating TB treatment outcomes is very important as it is an essential indicator of success in tuberculosis elimination [3]. The TB treatment success rate has reached 85% globally and 82% in the African region. The unsuccessfully treated cases are death, lost to follow up, failures, and not evaluated [1].

In Eritrea, the National TB and Leprosy Control Program is working with a vision of “Eritrea free of tuberculosis; zero deaths, disease and suffering due to TB for patients and families”. One of the program’s main objectives is to achieve TB treatment success rate ≥ 90% [4]. The program has been providing tuberculosis diagnosis and treatment for all TB patients free of charge [5]. As a result, 12,581 patients were treated for TB from 2014 to 2019. Furthermore, TB treatment success rate increased from 88% (2014) to 93% (2019), but 971 patients had unsuccessful treatment outcomes [6].

Unsuccessful TB treatment outcomes could be a challenge in controlling and eliminating TB. Several studies have explored factors that are associated with unsuccessful TB treatment outcomes. The common ones are sputum smear-negative TB case [7–10], having positive smear result at follow up [7, 9, 11], both pulmonary [12] and extrapulmonary TB [13], young [7] and old age [12, 14], HIV co-infection [7, 8, 13, 15], being male [12, 15], having had previous TB treatment [14–16], having diabetes co-morbidity [17, 18], and residing in rural areas [19, 20]. In addition, non-adherence to treatment was related to an increased risk of treatment failure, relapse and emergence of drug-resistant TB [21]. In contrast, young age, absences of drug resistance, not having previous TB treatment, higher education level and non-presence of co-morbidity were positively associated with successful TB treatment outcomes [22].

TB treatment success rate in Eritrea has reached national and global targets. However, death, treatment failure, lost to follow-up and not evaluated can threaten the gains made in controlling TB in this country. Especially, lost to follow up, not evaluated and treatment failure cases may remain infectious and could develop resistance and transmit the disease to the population. Factors associated with unsuccessful TB treatment outcomes should be explored and addressed to prevent further transmission and possible resistance. However, factors associated with unsuccessful TB treatment outcome remains unknown in this country. Therefore, this study explores the determinants of unsuccessful TB treatment outcomes in Eritrea’s Northern Red Sea region. This study’s results will fill the evidence gap and can be used to develop contextualized interventions. It can also serve as baseline data for future monitoring and further studies.

## Methods

### Study design

This study was a retrospective cohort study.

### Study setting and population

The study included all hospitals in the region: Afabet Hospital, Nakfa Hospital, Ghindae Regional Referral Hospital, and Massawa Hospital. They all fall under the second tier in healthcare delivery levels in Eritrea. One of them acts as a regional referral hospital (Ghindae Regional Referral Hospital), while three are called second contact hospitals. They have the same capacity in TB care and prevention services except Nakfa Hospital, which does not have a gene-x-pert machine for TB diagnosis. Thus, one sample from every TB presumptive is sent to the nearby hospital (Afabet Hospital) for gene-x-pert based TB diagnosis.

TB diagnosis in Eritrea follows the standard diagnostic procedures in which bacteriological confirmed TB are diagnosed by smear microscopy and Gene-Xpert MTB/RIF. In contrast, clinically diagnosis is done by X-ray in the presence of solid clinical suspicion by a physician. After a patient is diagnosed, a standard TB treatment regime of six months, divided into intensive and continuation phases, is initiated. The treatment is provided by a suitable direct observed treatment (DOT) provider either at the health facility or in a community close to patient’s residence. Patients are monitored at regular intervals to assess their response to the treatment with routine sputum smear microscopy test at the second, fifth and end of treatment for bacteriologically confirmed TB patients and clinical monitoring for those clinically diagnosed TB cases. At the end of TB treatment, patients’ files are closed by recording their treatment outcome at the TB treatment register, TB treatment card [5].

All patients registered for drug-susceptible TB treatment from January 2014 to December 2019 were the subjects of the current study. Patients with incomplete data, rifampicin-resistant/multidrug-resistant TB (RR/MDR-TB), and patients who had a diagnosis change (had no TB) were excluded from this study.

### Operational definition of variables

The following standard world health organization (WHO) definitions were adopted for operational use in the Eritrean national TB guidelines and in this study as well [23].

Cured: A pulmonary TB patient with bacteriologically confirmed TB at the beginning of treatment who was smear or culture-negative in the last month of treatment and on at least one previous occasion.

Treatment completed: A TB patient who completed treatment without evidence of failure but with no record to show that sputum smear or culture results in the last month of treatment and on at least one previous occasion were negative, either because tests were not done or because results are unavailable.

Treatment failed: A TB patient whose sputum smear or culture is positive in the 5^th^ month or later during treatment.

Died: A TB patient who dies for any reason before starting or during treatment.

Lost to follow up: A TB patient who did not start treatment or whose treatment was interrupted for 2 consecutive months or more.

Not evaluated: A TB patient for whom no treatment outcome is assigned. This includes cases “transferred out” to another treatment unit as well as cases for whom the treatment outcome is unknown to the reporting unit.

Treatment success: The sum of cured and treatment completed.

Unsuccessful treatment: treatment failure, lost to follow up, death and not evaluated are considered as unsuccessful TB treatment outcomes.

### Ethics approval and consent to participate

Ethical approval was obtained from the Health Research and Ethical committee of the Ministry of Health, Eritrea. The data was collected anonymously and did not include any personal identifiers to ensure confidentiality.

### Instrument and data collection

Data were collected from the TB treatment register, TB laboratory register and TB treatment cards available at the respective TB treatment sites. A data extraction tool designed for this purpose was used to collect the required data. This tool was first pretested by collecting data for 15 TB cases registered in 2013 in Massawa Hospital. The data extraction tools were modified to capture the necessary information depending on results of the pretest.

The final data extraction tool had two sections, with the first section containing information on socio-demographic characteristics of patients such as age, sex, residence, and treatment sites (health facility), and section two contains information on clinical variables such as type of patient, patient treatment category, laboratory results (smear microscopy), human immune deficiency virus (HIV) test results, anti-retroviral therapy (ART) and Co-trimoxazole preventive therapy (CPT) enrolment and TB treatment outcome.

### Variables

TB treatment outcome was the dependent variable in this study. TB treatment outcome decision is done by a physician or TB clinician based on the laboratory and or clinical findings as appropriate. The decision is made at the end of the treatment duration of six months for pulmonary TB and up to one year for some forms of exrapulmonary TB. In this study, cure and treatment complete were considered successful TB treatment outcomes. In contrast, treatment failure, lost to follow up, death and not evaluated were regarded as unsuccessful TB treatment outcomes. The socio-demographic and clinical variables include age, sex, residence, TB treatment site (hospital), history of TB treatment, type of TB, TB treatment category, smear microscopy result, HIV status, ART and CPT enrollment risk factors.

### Data analysis

After checking the completeness of the collected data and making logic checks (consistency), it was coded, doubled entered and analyzed using Stata version 13. Simple frequencies, percentages, proportions, mean and standard deviation were used to describe the data. To identify the determinants of treatment outcome, first univariate analysis was performed using the odds ratio as a measure of association with 95% confidence interval (CI) and p-values. All variables that were independently associated, p-value <0.2, with the outcome of interest (TB treatment outcome) in the univariate analysis were considered for the multivariable logistic regression model. Likelihood ratio statistic was used to build the final model by adding potential risk factors using forward selection with a highly associated variable. During model building process, variables that improved the model fit were retained, otherwise dropped. Thus, the final analysis was obtained based on the variables that were kept in the model. After variables that built the model were identified, a multicollinearity test was carried out; but no collinearity was detected.

## Result

### Study participants profile

**Fig 1:**
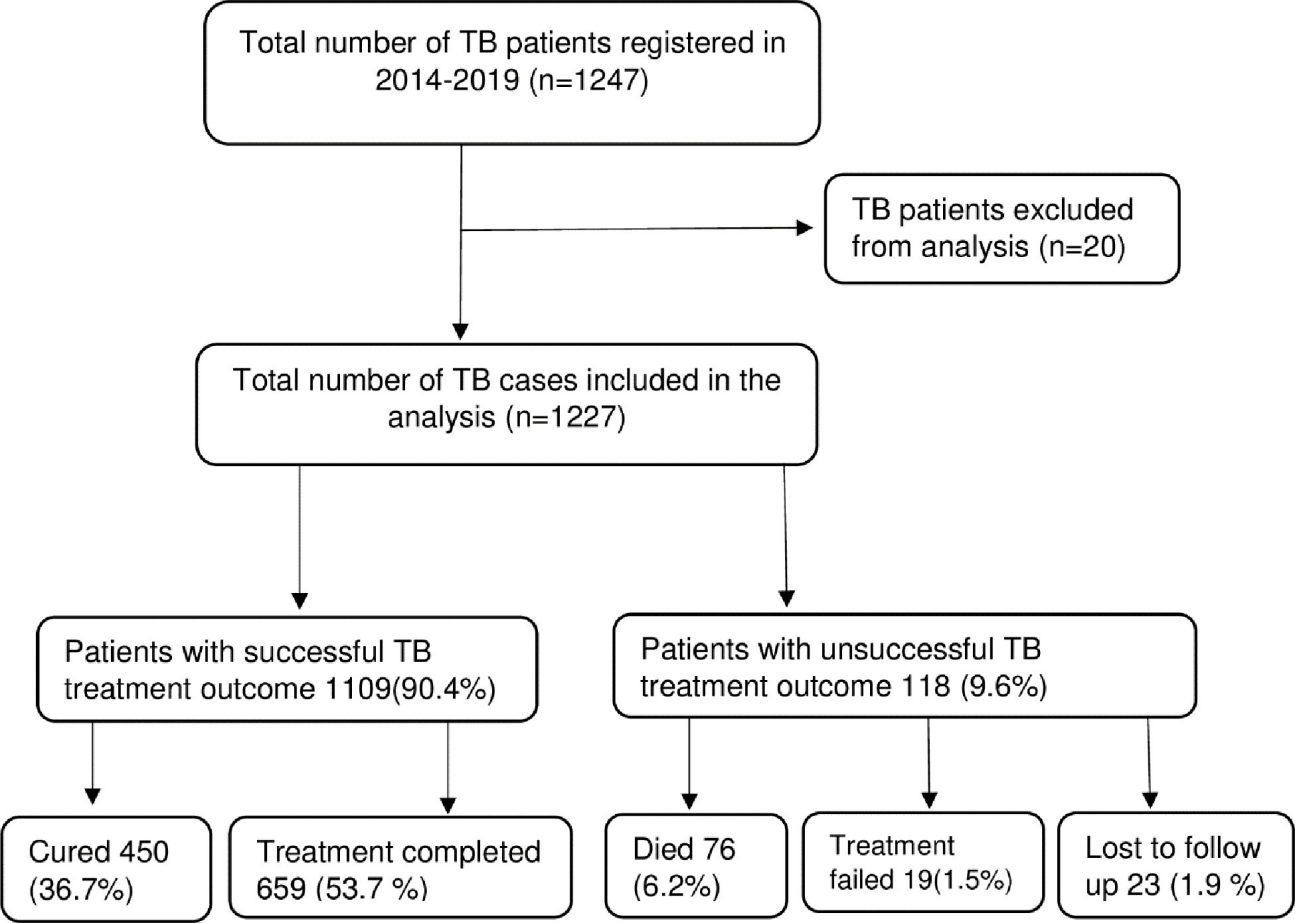
Consort chart showing cases selection for analysis and their TB treatment outcome in Northern Red Sea region, 2014–2019 cohort (n = 1227).

There were 1247 registered cases in the past six years (2014–2019) in the four hospitals in the Northern Red Sea region. TB patients with incomplete data, patients with RR/MDR-TB, and patients who had a change of diagnosis (had no TB) were not included in this analysis. Therefore, data for 1227 TB cases were finally analyzed for this study (Fig 1).

The majority (54.8%) of the TB cases were male patients, and 67.4% were between 15–54 years old, with a mean age of 36.5±18.9 years. Likewise, 57.6% of all TB cases were urban residents (Table 1). According to the history of TB treatment, almost all (94.3%) patients were new cases who did not have a previously TB treatment episode. Pulmonary TB was the common site of the disease in the majority (68%) of the cases. Results of the HIV test indicated that only 1.5% of TB cases were HIV positive (Table 1).

**Table 1:** Socio-demographic and clinical variables of study participants in Northern Red Sea region, 2014–2019 cohort (n = 1227).

Variable		Frequency	Percentage
**Sex (1225)**			
Male		671	54.8%
Female		554	45.2%
**Age range (1226)**			
≤ 14		161	13.1%
15–24		202	16.5%
25–34		198	16.2%
35–44		217	17.7%
45–54		208	17.0%
55–64		142	11.6%
≥ 65		98	8.0%
**Residence (1223)**			
Urban		705	57.6%
Rural		518	42.4%
**History of TB treatment**			
New		1157	94.3%
Previously treated	Relapse	66	5.4%
	Treatment after failure	2	0.2%
	Treatment after lost to follow up	2	0.2%
**Type of TB**			
Pulmonary TB (bacteriological confirmed)		502	40.9%
Pulmonary TB (clinical diagnosed)		332	27.1%
Extra-pulmonary TB (bacteriological confirmed)		21	1.7%
Extra-pulmonary TB (clinical diagnosed)		372	30.3%
**TB treatment category**			
Category-I		1014	82.6%
Category-II		70	5.7%
Category-III		143	11.7%
**HIV status (1226)**			
Negative		1208	98.5%
Positive		18	1.5%
**AFB sputum results at diagnosis**			
Positive		523	42.6%
Negative		318	25.9%
NA		386	31.5%
**AFB sputum results at 2**^**nd**^ **month(1224)**			
Positive		35	2.9%
Negative		741	60.5%
NA		448	36.6%
**Health facility**			
Afabet Hospital		410	33.4%
Ghindae Regional Referral Hospital		289	23.6%
Massawa Hospital		345	28.1%
Nakfa Hospital		183	14.9%

N.B. Discrepancy in numbers is due to missing values

### TB treatment outcome

Generally, 9.6% of the patients had unsuccessful TB treatment outcomes. Among those with unsuccessful TB treatment outcomes, 6.2% died, 1.9% lost to follow-up, and 1.5% had treatment failure (Fig 1).

The proportion of unsuccessful TB treatment increases as the age of TB patients increases, from 5% in the 15–24 age group to 21.4% in those ≥ 65. This difference was statistically significant (p < 0.001). Concerning the area of residence, there was no difference in TB treatment outcomes among urban (9.4%) and rural (9.6%) residents (Table 2).

**Table 2:** Comparison among successful and unsuccessful TB treatment outcomes in Northern Red Sea region, 2014–2019 cohort (n = 1227).

Variable	Successful TB treatment outcome	Unsuccessful TB treatment outcome,	p-value
**Sex**			
Male	614(91.5%)	57(8.5%)	0.17
Female	494(89.2%)	60(10.8%)	
**Age category**			
≤ 14	152(94.4%)	9(5.6%)	< 0.001
15–24	192(95%)	10(5%)	
25–34	181(91.4%)	17(8.6%)	
35–44	196(90.3%)	21(9.7%)	
45–54	190(91.4)	18(8.6%)	
55–64	121(85.2%)	21(14.8%)	
≥ 65	77(78.6%)	21(21.4%)	
**Residence**			
Urban	639(90.6%)	66(9.4%)	0.86
Rural	468(90.4%)	50(9.6%)	
**History of TB treatment**			
New	1050(90.8%)	107(9.2%)	.075
Previously treated	59(84.3%)	11(15.7%)	
**Type of TB**			
Pulmonary TB (bacteriological confirmed)	455(90.6%)	47(9.4%)	0.08
Pulmonary TB (clinical diagnosed)	291(87.6)	41(12.4%)	
Extra pulmonary TB	363(92.4%)	30(7.6%)	
**TB treatment category**			
Category-I	915(87.9%)	99(12.1%)	0.06
Category-II	59(84.3%)	11(15.7%)	
Category-III	135(94.4%)	8(5.6%)	
**HIV status**			
Negative	1097(90.8%)	111(9.2%)	< 0.001
Positive	11(61.1%)	7(38.9%)	
**Sputum result** at 2^nd^ month			
Positive	26(74.3%)	9(25.7%)	< 0.001
Negative	708(95.5%)	33(4.5%)	
NA	374(83.5%)	74(16.5%)	
**Health facility**			
Afabet Hospital	397(96.8%)	13(3.2%)	< 0.001
Ghindae Regional Referral Hospital	245(84.8%)	44(15.2%)	
Massawa Hospital	298(86.4%)	47(13.6%)	
Nakfa Hospital	169(92.4%)	14(7.6%)	

Among TB patients with a history of previous TB treatment, 15.7% had events of unsuccessful TB treatment outcomes. Similarly, 38.9% of HIV positive TB patients had unsuccessful TB treatment occurrence, which was statistically significant (p < 0.001). Similarly, among TB cases with positive sputum smear at 2^nd^ month of treatment, more than one fourth (25.7%) of them had unsuccessful TB treatment outcomes, which was statistically significant (p < 0.001). In terms of health facility, 15.2% of TB cases in Ghindae Regional Referral Hospital and 13.6% of TB cases in Massawa Hospital had unsuccessful TB treatment outcomes, and it was statistically significant (p <0.001) (Table 2).

### Determinants of unsuccessful TB treatment outcome

In multivariable logistic regression analysis, age, HIV status, and health facility were found to be independent predictors of unsuccessful TB treatment outcomes. TB cases 55–64 years old (AOR: 2.75[CI: 1.21–6.32], p = 0.016) and those ≥ 65 years old (AOR: 4.02[CI: 1.72–9.45], p = 0.001) had higher probability of unsuccessful TB treatment outcome. Besides, being HIV positive (AOR: 5.13[CI: 1.87–14.06], p = 0.002), being treated in Ghindae Regional Referral Hospital (AOR: 5.01[2.61–9.61], p < 0.001), Massawa Hospital (AOR: 4.35[2.28–8.30], p < 0.001) and Nakfa Hospital (AOR: 2.53[1.15–5.53], p = 0.021) were more likely to have unsuccessful TB treatment outcome (Table 3).

**Table 3:** Risk factors for unsuccessful TB treatment outcome in Northern Red Sea region, 2014–2019 cohort (n = 1227).

	Treatment outcome			
Variable	Successful TB treatment outcome	Unsuccessful TB treatment outcome	COR[CI,95%]	AOR[CI,95%]
**Sex**				
Male	614(91.5%)	57(8.5%)	1	
Female	494(89.2%)	60(10.8%)	1.31 [0.89–1.92]	
**Age category**				
≤ 14	152(94.4%)	9(5.6%)	1	1
15–24	192(95%)	10(5%)	0.88 [0.35–2.22]	0.72[0.28–1.87]
25–34	181(91.4%)	17(8.6%)	1.59 [0.69–3.66]	1.33[0.56–3.13]
35–44	196(90.3%)	21(9.7%)	1.81 [0.81–4.06]	1.56[0.68–3.56]
45–54	190(91.4)	18(8.6%)	1.60 [0.70–3.66]	1.44[0.62–3.34]
55–64	121(85.2%)	21(14.8%)	2.93[1.30–6.63]**	2.76[1.21–6.32]*
≥ 65	77(78.6%)	21(21.4%)	4.61[2.01–10.54]***	4.03[1.72–9.45]**
**Residence**				
Urban	639(90.6%)	66(9.4%)	1	
Rural	468(90.4%)	50(9.6%)	0.97[0.66–1.42]	
**History of TB treatment**				
New	1050(90.8%)	107(9.2%)	1	
Previously treatment	59(84.3%)	11(15.7%)	1.83[0.93–3.59]	
**Type of TB**				
Pulmonary TB (bacteriological confirmed)	455(90.6%)	47(9.4%)	1.25[0.78–2.02]	
Pulmonary TB (clinical diagnosed)	291(87.6)	41(12.4%)	1.71[1.04–2.80]*	
Extra pulmonary TB	363(92.4%)	30(7.6%)	1	1
**TB treatment category**				
Category-I	915(87.9%)	99(12.1%)	1.83[0.87–3.84]	1.17[0.17–7.90]
Category-II	59(84.3%)	11(15.7%)	3.15[1.20–8.22]*	2.16[0.28–16.73]
Category-III	135(94.4%)	8(5.6%)	1	1
**HIV status**				
Negative	1097(90.8%)	111(9.2%)	1	1
Positive	11(61.1%)	7(38.9%)	6.29[2.39–16.55]***	5.13[1.85–13.89]**
**Sputum result** at 2^nd^ month				
Positive	26(74.3%)	9(25.7%)	1	
Negative	708(95.5%)	33(4.5%)	0.14[0.06–0.31]***	
NA	374(83.5%)	74(16.5%)	0.57 [0.26–1.27]	
**Health facility**				
Afabet Hospital	397(96.8%)	13(3.2%)	1	
Ghindae Regional Referral Hospital	245(84.8%)	44(15.2%)	5.48[2.90–10.39]***	5.01[2.61–9.61]***
Massawa Hospital	298(86.4%)	47(13.6%)	4.82[2.56–9.06]***	4.35[2.28–8.30]***
Nakfa Hospital	169(92.4%)	14(7.6%)	2.53[1.16–5.50]*	2.53[1.15–5.53]*

N.B. P< 0.05(*), P< 0.01(**), P< 0.001(***)

## Discussion

This study revealed that 9.6% of all TB patients in the Northern Red Sea region had unsuccessful TB treatment outcomes during 2014–2019. This result is 1.1% lower than the average unsuccessful TB treatment success rate of 8.5% at the national level [1]; but meets the global target for TB treatment success rate [24]. Several studies reported similar rates [10, 25, 26], whereas others reported higher rates of unsuccessful TB treatment outcomes [8, 9, 27, 28]. The probable reasons for the variation in the rates of unsuccessful TB treatment outcomes among nations could be the difference in healthcare capacity to treat appropriately, support and monitor TB patients, TB burden and other co-morbidities, and coverage of treatment with anti-retroviral to those with HIV and others with respective co-morbidities.

In this study, older age (≥ 55) was one of the independent predictors of unsuccessful TB treatment outcome. This is consistent with the findings of numerous studies which reported an association between old age with unsuccessful TB treatment outcomes [8, 9, 26, 29]. On the contrary, one study found unsuccessful TB treatment outcomes to be common in young TB patients [30]. There were also other reports in which both young and old TB patients had unsuccessful TB treatment outcomes [11, 31]. It’s well-known that older age TB patients may have age-related weakened immunity and an increased number of chronic co-morbidities, contributing to the risk of unsuccessful TB treatment outcomes. But in this study, all TB patients above 55 years old were HIV negative which rules out HIV as a possible reason behind the unsuccessful TB treatment outcomes in this specific age group. Older TB patients may also have atypical signs and symptoms, making TB diagnosis difficult. This could lead to delayed diagnosis and treatment initiation and increase TB-related morbidity and mortality. Furthermore, TB treatment in elderly patients is challenging due to an increased risk of adverse drug reactions [32].

In the current study, HIV positive TB patients had a higher likelihood of unsuccessful TB treatment outcomes. This agrees to prior studies that reported higher odds of unsuccessful TB treatment outcomes among HIV positive TB patients [7, 19, 33–35] but there were also reports in which unknown HIV status was a determinant of unsuccessful TB treatment outcomes [10, 25]. In Eritrea, The implementation of TB-HIV integrated services is well established. All TB patients and TB presumptive are routinely offered HIV counselling and testing as part of these integrated services. HIV positives cases are also linked to anti-retroviral therapy (ART) services. Similarly, all people living with HIV are regularly screened for TB, and if found to have TB, they are linked to TB services. As a result, in this study, 94.4% of HIV positive TB patients were enrolled on ART and co-trimoxazole preventive therapy (CPT). Nevertheless, 35.3% of them had unsuccessful TB treatment outcomes. One of the possible reasons could be that patients may already have developed an advanced disease at presentation. This could be due to possible late healthcare seeking by patients, and late diagnosis of the disease, which could arise from difficulties in diagnosing TB, as patients who have TB-HIV co-infection may have altered clinical presentations [36, 37]. In a patient with an advanced HIV infection, anti-mycobacterium medications have low bioavailability due to malabsorption of anti-TB drugs [38]. Besides, concurrent treatment of TB-HIV is related to high pill load, drug-drug interactions (between some anti-TB and anti-retroviral medications), coinciding and numerous toxic effects and drug intolerance [39], which could lead to treatment interruption, non-adherence [36, 39, 40] and consequent unsuccessful TB treatment outcome.

The health facility was also a predictor of unsuccessful TB treatment outcomes. In line with our findings, previous studies reported the association of health facility facilities with unsuccessful TB treatment outcomes [9, 25, 41]. Ghindae Regional Referral Hospital, Massawa Hospital, and Nakfa Hospital had higher odds of unsuccessful TB treatment outcomes. In our context, most critically ill patients are referred from lower-level health facilities to hospitals for better diagnosis and treatment. Despite the referral of these critical cases, hospitals could have a high workload due to the variety of available healthcare services. Thus, patients may not receive very individualized care and support. In addition, the referral by itself could be late and the disease could have been already complicated. Another important scenario is that patients referred from lower health facilities may not stay for the required admission period. Hence, they could interrupt treatment, leading to unsuccessful TB treatment outcomes.

## Conclusion

This study discovered that the proportion of unsuccessful TB treatment outcomes in the Northern Red Sea region is low. Old age, HIV co-infection and health facility were the independent predictors of unsuccessful TB treatment outcomes. Therefore, to reduce unsuccessful TB treatment outcomes, the program should implement risk factors focused intervention such as providing screening and treatment for the prevalent chronic comorbid conditions in older age TB patients, addressing any barriers to early diagnosis and treatment of patients with a special focus on HIV positives, and introduction of special care and support for TB cases who are in need.

### Study limitation

Due to the nature of the study, data was collected retrospectively from existing TB registers and medical records. Thus, some information, such as co-morbidities other than HIV/AIDS were missing, and thus our results should be interpreted accordingly.

## Supporting information

S1 Table Data collection tool for determinants of unsuccessful TB treatment outcome in Northern Red Sea region, Eritrea. (PDF)

## Abbreviations

AFB: Acid-fast bacilli
AOR: adjusted odds ratio
ART: anti-retroviral therapy
BMI: body mass index
CI: confidence interval
COR: crude odds ratio
CPT: Co-trimoxazole preventive therapy
DM: diabetes mellitus
FBG: fasting blood glucose
FBS: fasting blood sugar
HbA1c: glycosylated haemoglobin
HIV/AIDS: human immune deficiency virus/ Acquired Immune Deficiency Syndrome
HMIS: health management information system
IDF: international diabetes federation
IUATLD: international union against TB and lung disease
IQR: interquartile ranges
MOH: ministry of health
MTB: mycobacterium tuberculosis
RIF: rifampicin
TB: tuberculosis
WHO: world health organization
