# Supplementary material for: Determinants of unsuccessful tuberculosis treatment outcome in Northern Red Sea region, Eritrea
Source: PLoS One. 2022 Aug 15;17(8):e0273069. doi: 10.1371/journal.pone.0273069 (PMC9377576; doi:10.1371/journal.pone.0273069)
Supplement: S1 Table — (PDF) [file pone.0273069.s001.pdf]

Determinants of unsuccessful TB treatment outcome

Data extraction tool

**Name of health facility**				
**Year of treatment**				
**Questionnaire Code**				

Please circle the numbers/ fill your response in the answer space provided.			
**SECTION 1: DEMOGRAPHIC DATA**			
**NO**	**QUESTIONS AND FILTERS**	**CODING CATAGORIES**	
101	Age in completed years	…………………………….............<br>Missing…………………………..999	
102	Sex	Female………………………………………..1<br>Male…………………………………………..2<br>Missing………………………………………...999	
103	Permanent residence	Urban …………………………………………1<br>Rural …………………………………………2<br>Missing………………………………………999	
**SECTION 2:CLINICAL INFORMATION**			
201	Type of TB patient (registration group)	New ……………………………………………1<br>Relapse ………………………………………2<br>Failure…………………………………………..3<br>Treatment after lost to follow up………………….4<br>Other previously treated..…………………………5<br>Unknown history…………………………………6<br>Missing………………………………………..999	
202	TB site and diagnosis	Pulmonary (Bacteriological diagnosed)……….………1<br>Pulmonary (clinical diagnosed)…………………….….2<br>Extrapulmonary (Bacteriological diagnosed)……………....3<br>Extrapulmonary (clinical diagnosed)………………………4<br>Missing…………………………………………………999	
203	Treatment category	Cat I……………………………………………1<br>Cat II…...2<br>Cat III…………………………………………..3<br>Missing………………………………………...999	
204	Sputum smear result (AFB microscopy)	Baseline/diagnosis	Positive…………………..1<br>Negative…………………2<br>Missing…………………999<br>NA……………………..777
		2nd month	Positive…………………..1<br>Negative…………………2<br>Missing…………………999<br>NA……………………..777
		5th month	Positive…………………..1<br>Negative…………………2<br>Missing…………………999<br>NA……………………..777
		End of treatment	Positive…………………..1<br>Negative…………………2<br>Missing…………………999<br>NA……………………..777

205	Body weight (kg)	Baseline ………………………Kg<br>End of treatment………………Kg<br>Missing……………………..999
206	HIV test result	Negative…………………….……………………..1<br>Positive……………………………………………2<br>Unknown…………………………………………..3
206(a)	*For HIV positive only,*<br>*Is Patient on ART*	Yes…………………………………………………1<br>No…………………………………………………2
206(b)	*For HIV positive only,*<br>*Is Patient on CPT*	Yes…………………………………………………1<br>No…………………………………………………2
207	Treatment outcome	Cure……………………………………………..1<br>Complete………………………………………..2<br>Failure…………………………………………..3<br>Died……………………………......................4<br>Lost to follow up………………………………..5<br>Not evaluated……………………………………6

--The End--

Determinants of unsuccessful TB treatment outcome

Operational definitions for study variables

Pulmonary tuberculosis (PTB): refers to any bacteriologically confirmed or clinically diagnosed case of TB involving the lung parenchyma or the tracheobronchial tree. A patient with both pulmonary and extrapulmonary TB should be classified as a case of PTB.

Extrapulmonary tuberculosis (EPTB): refers to any bacteriologically confirmed or clinically diagnosed case of TB involving organs other than the lungs, e.g. pleura, lymph nodes, abdomen, genitourinary tract, skin, joints and bones, meninges.

Bacteriologically confirmed TB case: is one from whom a biological specimen is positive by smear microscopy, culture, Xpert MTB/RIF.

Clinically diagnosed TB case: is one who does not fulfil the criteria for bacteriological confirmation but has been diagnosed with active TB by a clinician or other medical practitioner who has decided to give the patient a full course of TB treatment. This definition includes cases diagnosed on the basis of X-ray abnormalities or suggestive histology and extrapulmonary cases without laboratory confirmation.

New: patients have never been treated for TB or have taken anti-TB drugs for less than 1 month.

Previously treated: patients have received 1 month or more of anti-TB drugs in the past. They are further classified by the outcome of their most recent course of treatment:

> **Relapse:** patients have previously been treated for TB, were declared cured or treatment completed at the end of their most recent course of treatment, and are now diagnosed with a recurrent episode of TB (either a true relapse or a new episode of TB caused by reinfection).
>
> **Treatment after failure:** patients are those who have previously been treated for TB and whose treatment failed at the end of their most recent course of treatment.
>
> **Treatment after loss to follow-up:** patients have previously been treated for TB and were declared lost to follow-up at the end of their most recent course of treatment. (These were previously known as treatment after default patients.)

Cat I treatment regimen: new TB cases are given the cat I treatment regimen. The intensive phase consists of 2 months of Isoniazid, Rifampicin, Pyrazinamide and Ethambutol given daily under direct observation. The continuation phase consists of 4 months of Isoniazid and Rifampicin given daily under direct supervision.

Cat II treatment regimen: retreatment cases are given the cat II treatment regimen. The intensive phase consists of total 3 months; first two months with Isoniazid, Rifampicin, Pyrazinamide, Ethambutol and Streptomycin followed by one month of INH, Rifampicin, Pyrazinamide and Ethambutol. The continuation phase consists of 5 months of Isoniazid, Rifampcin and Ethambutol. According to the lates WHO guideline there is no more Cat II and patients who used to be treated in this group shoulbe be treated similar to Cat I.

Cat III treatment regimen: children are given cat III treatment regimen. The intensive phase consist of total of 2 months of Isoniazid, Rifampicin, Pyrazinamide, with or without Ethambutol based on the severity and HIV infection of the patient. The continuation phase consists of 4 months of Isoniazid and Rifampicin given daily under direct supervision.

Determinants of unsuccessful TB treatment outcome

HIV-positive TB: patient refers to any bacteriologically confirmed or clinically diagnosed case of TB who has a positive result from HIV testing conducted at the time of TB diagnosis or other documented evidence of enrolment in HIV care, such as enrolment in the pre-ART register or in the ART register once ART has been started.

HIV-negative TB: patient refers to any bacteriologically confirmed or clinically diagnosed case of TB who has a negative result from HIV testing conducted at the time of TB diagnosis. Any HIV-negative TB patient subsequently found to be HIV-positive should be reclassified accordingly.

HIV status unknown TB: patient refers to any bacteriologically confirmed or clinically diagnosed case of TB who has no result of HIV testing and no other documented evidence of enrolment in HIV care. If the patient's HIV status is subsequently determined, he or she should be reclassified accordingly.
